# Treatment of Simple Ulcer of the Stomach

**Published:** 1871-04

**Authors:** H. Ziemssen

**Affiliations:** Professor of Clinical Medicine in the University of Erlangen, Bavaria


ART. IV.—*Treatment of Simple Ulcer of the Stomach.* By H ZIEMSSEN, Professor of Clinical Medicine in the University of Erlangen, Bavaria.

(From collection of Clinical Lectures in combination with German Clinical Professors, edited by Richard Volkman, Professor of Clinical Surgery in the University of Halle, Prussia, V. 15, 25th January, 1871.)

TRANSLATED BY GEO. NIEMEIER, M. D., FOR THE "BUFFALO MEDICAL AND SURGICAL JOURNAL."

WUERZBURG, Bavaria.

GENTLEMEN,—The pathology of the simple ulcer of the stomach has been, in the last ten years, the subject of careful studies, which have enriched our knowledge of the commencement and course of the ulcerative process; the same cannot be said about the therapeutics of it. We must confess that, regarding the treatment, we are, in the main points, yet following Cruveilhier and Authenrieth. Yet our present knowledge of the pathological and physiological processes of the ulcer of the stomach, and clinical experience, allow us a more definite conception of the indications, and a rational empirical therapia. I might assert that there is hardly another affection which combines, with equal clearness of the results to be attained and the ways leading to it, or the same certainty of obtaining the aim. Before we turn our attention to the patho-physiological processes, I will briefly mention those movements to which, according to the present standing of our knowledge, are ascribed the most prominent causes for the ulcer of the stomach: they are especially changes of the vessels of the stomach, which restrict or arrest the circulation within a circumscribed spot of the membranes, and by it expose this spot to the corroding influence of the gastric juice, to self-digestion. Virchow, Rokitansky, and Nerkel, have proved, on fresh ulcers, that the lumen of small arteries can be entirely closed by embolism or thrombosis, by fatty, atheromatous and amyloid degeneration of their walls; and Pavy experimentally proved its confirmation, in causing ulcers by ligaturing arterial branches of the stomach in animals. It is further very probable that circulatory disturbances of another kind, for example, intense imflammatory or collateral hyperæmia, such as extensive burns of the skin, or venous stagnation, in consequence of mechanical impediments in the course of the vena, portaram can lead to serious disturbances in circumscribed spots of the stomach wall, as the tearing of the smallest vessels causes a hemorrhagic infiltration of the mucous membrane. Whether the nutrition of a mucous spot has suffered by arrest of circulation or by hemorrhagic infiltration, in both cases the spot of the mucous membrane is exposed to the corroding influence of the gastric juice, or to the sharply acid products of an anamalous digestion, and now a superficial necrosis may follow, at first of the mucous membrane; and if the nutrition is disturbed to a larger extent in its depth, at last a necrosis of the other tissues of the stomach wall. It is further possible that, in some cases, a simple erasion, a catarrhal ulcer, or a diptheritic infiltration, may cause a round ulcer. We see, therefore, the loss of substance, which we call ulcer of the stomach, can be the result of quite heterogeneous pathological processes in the stomach wall, and is void of any specification. Regarding its disposition towards the sexes, it is a fact that at least double as many women as men suffer with it.

(TO BE CONTINUED.)

